# Supplementary material for: OpenApePose, a database of annotated ape photographs for pose estimation
Source: eLife. 2023 Dec 11;12:RP86873. doi: 10.7554/eLife.86873 (PMC10712952; doi:10.7554/eLife.86873)
Supplement: Supplementary file 1. [file elife-86873-supp1.docx]

**Supplementary File 1:** Datasheet for the OpenApePose dataset.

**Datasheet for the OpenApePose dataset**

**Motivation**

1. **For what purpose was the dataset created?**

The dataset was created to enable training pose estimation models for non-human apes. Such models allow automated pose tracking and behavior monitoring, which greatly enhances the resolution and quantity of behavioral data that researchers can use. Automated pose tracking in non-human primates has applications in a wide range of fields, such as primatology, neuroscience, and disease research.

1. **Who created the dataset (for example, which team, research group) and on behalf of which entity (for example, company, institution, organization)?**

The dataset was created in collaboration with researchers from the University of Minnesota, Twin Cities (Nisarg Desai, Praneet Bala, Jan Zimmermann, and Benjamin Hayden), and Emory University (Rebecca Richardson, Jessica Raper).

1. **Who funded the creation of the dataset?**

This work was supported by NIH MH128177 (to JZ), P30 DA048742 (JZ, BH), MH125377 (BH), NSF 2024581 (JZ, BH) and a UMN AIRP award (JZ, BH) from the Digital Technologies Initiative (JZ, BH), from the Minnesota Institute of Robotics (JZ), and Emory National Primate Research Center, NIH Office of the Director (P51-OD011132).

1. **Any other comments?**

More details in the manuscript and on Github at: https://github.com/desai-nisarg/OpenApePose

**Composition**

1. **What do the instances that comprise the dataset represent (for example, documents, photos, people, countries)?**

OpenApePose contains 71,868 photographs, annotated with 16 body landmarks, of six ape species in naturalistic contexts.

1. **How many instances are there in total (of each type, if appropriate)?**

The table below describes number of photos from each species included in the dataset.

| **Species** | Bonobos | Chimpanzees | Gibbons | Gorillas | Orangutans | Siamangs |
| --- | --- | --- | --- | --- | --- | --- |
| **Images** | 11,685 | 18,010 | 9,274 | 12,905 | 12,722 | 7,272 |

1. **What data does each instance consist of?**

Each instance consists of an image that would have at least one ape individual.

1. **Is there a label or target associated with each instance?**

Each image has an associated JSON entry that includes the filename, the species name, a bounding box around an individual ape, the coordinates of 16 body landmarks in the following order: Nose, Left eye, Right eye, Head, Neck, Left shoulder, Left elbow, Left wrist, Right shoulder, Right elbow, Right wrist, Hip/Sacrum, Left knee, Left foot, Right knee, Right foot, and an entry specifying whether each landmark is visible or not.

1. **Is any information missing from individual instances?**

All instances include the aforementioned details.

1. **Are relationships between individual instances made explicit (for example, users' movie ratings, social network links)?**

No

1. **Are there recommended data splits (for example, training, development/validation, testing)?**

We include an example split to train our model and demonstrate the utility of the dataset. However, users should feel free to use their own splits to train better models.

1. **Are there any errors, sources of noise, or redundancies in the dataset?**

We have looked at each instance separately to minimize any errors and provide high-quality annotations. However, given the size of the dataset, it is possible that some errors were missed. We encourage users to report any errors they find on the GitHub page.

1. **Is the dataset self-contained, or does it link to or otherwise rely on external resources (for example, websites, tweets, other datasets)?**

The dataset is self-contained.

1. **Does the dataset contain data that might be considered confidential (for example, data that is protected by legal privilege or by doctor-patient confidentiality, data that includes the content of individuals' non-public communications)?**

No.

1. **Does the dataset contain data that, if viewed directly, might be offensive, insulting, threatening, or might otherwise cause anxiety?**

No.

**Preprocessing/cleaning/labeling**

1. **Was any preprocessing/cleaning/labeling of the data done (for example, discretization or bucketing, tokenization, part-of-speech tagging, SIFT feature extraction, removal of instances, processing of missing values)?**

The images included in the dataset are a subset of all the images taken for creating the dataset. We manually removed images that were repetitive, for example in cases when the ape was at rest and did not change the pose and in cases when extracting frames from videos. We also removed images that were too blurry to identify the body landmarks for human annotators.

1. **Was the "raw" data saved in addition to the preprocessed/cleaned/labeled data (for example, to support unanticipated future uses)?**

The raw images are saved and available upon request.

1. **Is the software that was used to preprocess/clean/label the data available?**

The images were manually chosen and deleted.

1. **Any other comments?**

No.

**Uses**

1. **Has the dataset been used for any tasks already?**

No.

1. **Is there a repository that links to any or all papers or systems that use the dataset?**

We do not have one yet. However, we will add a section on the GitHub page as others start to utilize the dataset.

1. **What (other) tasks could the dataset be used for?**

The dataset can be used for training action recognition models that can be used to track behavior. There are wide ranging applications of such models in fields such as primatology, neuroscience, disease research etc. Primatologists can use the dataset to train models to track ape behavior in the wild using camera traps or drones. Combining with individual recognition, such models could be used to automate many tasks in primate behavior research such as ethogramming, dominance hierarchy estimation etc. Neuroscientists can use this dataset to train models to track behavior in captivity and combine it with neurological measurements such as electrophysiology. In disease research, such models could be used to track behavior and motor changes during and after infections.

1. **Is there anything about the composition of the dataset or the way it was collected and preprocessed/cleaned/labeled that might impact future uses?**

As most images are from zoos and sanctuaries, the backgrounds and poses may not be representative of the range of specific conditions in which one may encounter individuals in the wild. Adding more images from wild settings may enhance the performance of future models.

1. **Are there tasks for which the dataset should not be used?**

No.

1. **Any other comments?**

No.

**Distribution**

1. **Will the dataset be distributed to third parties outside of the entity (for example, company, institution, organization) on behalf of which the dataset was created?**

The dataset is available for the public under MIT CC-BY-4.0 license.

1. **How will the dataset be distributed (for example, tarball on website, API, GitHub)?**

The dataset is available on Github at: https://github.com/desai-nisarg/OpenApePose

1. **When will the dataset be distributed?**

It is available now.

1. **Will the dataset be distributed under a copyright or other intellectual property (IP) license, and/or under applicable terms of use (ToU)?**

The dataset is available for the public under MIT CC-BY-4.0 license.

1. **Have any third parties imposed IP-based or other restrictions on the data associated with the instances?**

No.

1. **Do any export controls or other regulatory restrictions apply to the dataset or to individual instances?**

No.

1. **Any other comments?**

No.

**Maintenance**

1. **Who will be supporting/hosting/maintaining the dataset?**

The dataset will be maintained by Dr. Nisarg Desai. Any updates will be included on the GitHub page: <https://github.com/desai-nisarg/OpenApePose>

1. **How can the owner/curator/manager of the dataset be contacted (for example, email address)?**

Dr. Nisarg Desai can be contacted at nisarg [dot] p [dot] desai [at] gmail [dot] com

1. **Is there an erratum?**

We do not have one yet. However, we will add a section on the GitHub page as others start to utilize the dataset. We encourage users to report any errors they find.

1. **If others want to extend/augment/build on/contribute to the dataset, is there a mechanism for them to do so?**

The authors would be happy to augment the dataset. We encourage anyone who wishes to contribute to get in touch with Dr. Nisarg Desai and start a pull request on the GitHub page.

1. **Any other comments?**

No.
