# Supplementary material for: OpenApePose, a database of annotated ape photographs for pose estimation
Source: eLife. 2023 Dec 11;12:RP86873. doi: 10.7554/eLife.86873 (PMC10712952; doi:10.7554/eLife.86873)
Supplement: Supplementary file 2. — (a) Pairwise t-tests comparing the AUC values for different training and testing set combinations. AUCs were obtained across 100 random samples of 500 images from the test sets. p-Values adjusted for multiple comparisons using Bonferroni correction. ** not significant. (b) Pairwise t-tests comparing the PCK@0.2 values for different training and testing set combinations. PCK@0.2 values were obtained across 100 random samples of 500 images from the test sets. p-Values adjusted for multiple comparisons using Bonferroni correction. ** not significant [file elife-86873-supp2.docx]

**Supplementary File 2a:** Pairwise t-tests comparing the AUC values for different training and testing set combinations. AUCs were obtained across 100 random samples of 500 images from the test sets. P-values adjusted for multiple comparisons using Bonferroni correction. ** not significant

| Experiment 1 | Experiment 2 | Mean 1 | Mean 2 | difference | p.adj |
| --- | --- | --- | --- | --- | --- |
| OMP (no Apes) on OMP | OAP (100%) on OAP | 0.929 | 0.897 | 0.031 | 0 |
| OMP (no Apes) on OMP | OAP (90%) on OAP | 0.929 | 0.903 | 0.026 | 0 |
| OMP (no Apes) on OMP | OAP (70%) on OAP | 0.929 | 0.899 | 0.03 | 0 |
| OMP (no Apes) on OMP | OAP (50%) on OAP | 0.929 | 0.842 | 0.087 | 0 |
| OMP (no Apes) on OMP | OAP (30%) on OAP | 0.929 | 0.824 | 0.105 | 0 |
| OMP (no Apes) on OMP | OAP (10%) on OAP | 0.929 | 0.755 | 0.174 | 0 |
| OMP (no Apes) on OMP | OAP (100%) on OMP | 0.929 | 0.736 | 0.193 | 0 |
| OMP (no Apes) on OMP | OMP (no Apes) on OAP | 0.929 | 0.743 | 0.186 | 0 |
| OMP (no Apes) on OMP | COCO on OMP | 0.929 | 0.578 | 0.351 | 0 |
| OMP (no Apes) on OMP | COCO on OAP | 0.929 | 0.71 | 0.219 | 0 |
| OMP (no Apes) on OMP | COCO on COCO | 0.929 | 0.958 | -0.029 | 0 |
| OAP (100%) on OAP | OAP (90%) on OAP | 0.897 | 0.903 | -0.006 | 0 |
| OAP (100%) on OAP | OAP (70%) on OAP | 0.897 | 0.899 | -0.001 | 1** |
| OAP (100%) on OAP | OAP (50%) on OAP | 0.897 | 0.842 | 0.055 | 0 |
| OAP (100%) on OAP | OAP (30%) on OAP | 0.897 | 0.824 | 0.073 | 0 |
| OAP (100%) on OAP | OAP (10%) on OAP | 0.897 | 0.755 | 0.143 | 0 |
| OAP (100%) on OAP | OAP (100%) on OMP | 0.897 | 0.736 | 0.161 | 0 |
| OAP (100%) on OAP | OMP (no Apes) on OAP | 0.897 | 0.743 | 0.155 | 0 |
| OAP (100%) on OAP | COCO on OMP | 0.897 | 0.578 | 0.32 | 0 |
| OAP (100%) on OAP | COCO on OAP | 0.897 | 0.71 | 0.188 | 0 |
| OAP (100%) on OAP | COCO on COCO | 0.897 | 0.958 | -0.06 | 0 |
| OAP (90%) on OAP | OAP (70%) on OAP | 0.903 | 0.899 | 0.005 | 0 |
| OAP (90%) on OAP | OAP (50%) on OAP | 0.903 | 0.842 | 0.061 | 0 |
| OAP (90%) on OAP | OAP (30%) on OAP | 0.903 | 0.824 | 0.079 | 0 |
| OAP (90%) on OAP | OAP (10%) on OAP | 0.903 | 0.755 | 0.149 | 0 |
| OAP (90%) on OAP | OAP (100%) on OMP | 0.903 | 0.736 | 0.167 | 0 |
| OAP (90%) on OAP | OMP (no Apes) on OAP | 0.903 | 0.743 | 0.161 | 0 |
| OAP (90%) on OAP | COCO on OMP | 0.903 | 0.578 | 0.326 | 0 |
| OAP (90%) on OAP | COCO on OAP | 0.903 | 0.71 | 0.193 | 0 |
| OAP (90%) on OAP | COCO on COCO | 0.903 | 0.958 | -0.054 | 0 |
| OAP (70%) on OAP | OAP (50%) on OAP | 0.899 | 0.842 | 0.056 | 0 |
| OAP (70%) on OAP | OAP (30%) on OAP | 0.899 | 0.824 | 0.074 | 0 |
| OAP (70%) on OAP | OAP (10%) on OAP | 0.899 | 0.755 | 0.144 | 0 |
| OAP (70%) on OAP | OAP (100%) on OMP | 0.899 | 0.736 | 0.162 | 0 |
| OAP (70%) on OAP | OMP (no Apes) on OAP | 0.899 | 0.743 | 0.156 | 0 |
| OAP (70%) on OAP | COCO on OMP | 0.899 | 0.578 | 0.321 | 0 |
| OAP (70%) on OAP | COCO on OAP | 0.899 | 0.71 | 0.189 | 0 |
| OAP (70%) on OAP | COCO on COCO | 0.899 | 0.958 | -0.059 | 0 |
| OAP (50%) on OAP | OAP (30%) on OAP | 0.842 | 0.824 | 0.018 | 0 |
| OAP (50%) on OAP | OAP (10%) on OAP | 0.842 | 0.755 | 0.087 | 0 |
| OAP (50%) on OAP | OAP (100%) on OMP | 0.842 | 0.736 | 0.106 | 0 |
| OAP (50%) on OAP | OMP (no Apes) on OAP | 0.842 | 0.743 | 0.1 | 0 |
| OAP (50%) on OAP | COCO on OMP | 0.842 | 0.578 | 0.264 | 0 |
| OAP (50%) on OAP | COCO on OAP | 0.842 | 0.71 | 0.132 | 0 |
| OAP (50%) on OAP | COCO on COCO | 0.842 | 0.958 | -0.115 | 0 |
| OAP (30%) on OAP | OAP (10%) on OAP | 0.824 | 0.755 | 0.07 | 0 |
| OAP (30%) on OAP | OAP (100%) on OMP | 0.824 | 0.736 | 0.088 | 0 |
| OAP (30%) on OAP | OMP (no Apes) on OAP | 0.824 | 0.743 | 0.082 | 0 |
| OAP (30%) on OAP | COCO on OMP | 0.824 | 0.578 | 0.247 | 0 |
| OAP (30%) on OAP | COCO on OAP | 0.824 | 0.71 | 0.114 | 0 |
| OAP (30%) on OAP | COCO on COCO | 0.824 | 0.958 | -0.133 | 0 |
| OAP (10%) on OAP | OAP (100%) on OMP | 0.755 | 0.736 | 0.019 | 0 |
| OAP (10%) on OAP | OMP (no Apes) on OAP | 0.755 | 0.743 | 0.012 | 0 |
| OAP (10%) on OAP | COCO on OMP | 0.755 | 0.578 | 0.177 | 0 |
| OAP (10%) on OAP | COCO on OAP | 0.755 | 0.71 | 0.045 | 0 |
| OAP (10%) on OAP | COCO on COCO | 0.755 | 0.958 | -0.203 | 0 |
| OAP (100%) on OMP | OMP (no Apes) on OAP | 0.736 | 0.743 | -0.006 | 0 |
| OAP (100%) on OMP | COCO on OMP | 0.736 | 0.578 | 0.159 | 0 |
| OAP (100%) on OMP | COCO on OAP | 0.736 | 0.71 | 0.026 | 0 |
| OAP (100%) on OMP | COCO on COCO | 0.736 | 0.958 | -0.221 | 0 |
| OMP (no Apes) on OAP | COCO on OMP | 0.743 | 0.578 | 0.165 | 0 |
| OMP (no Apes) on OAP | COCO on OAP | 0.743 | 0.71 | 0.033 | 0 |
| OMP (no Apes) on OAP | COCO on COCO | 0.743 | 0.958 | -0.215 | 0 |
| COCO on OMP | COCO on OAP | 0.578 | 0.71 | -0.132 | 0 |
| COCO on OMP | COCO on COCO | 0.578 | 0.958 | -0.38 | 0 |
| COCO on OAP | COCO on COCO | 0.71 | 0.958 | -0.248 | 0 |

**Supplementary File 2b:** Pairwise t-tests comparing the PCK@0.2 values for different training and testing set combinations. PCK@0.2 values were obtained across 100 random samples of 500 images from the test sets. P-values adjusted for multiple comparisons using Bonferroni correction. ** not significant

| Experiment 1 | Experiment 2 | Mean 1 | Mean 2 | difference | p.adj |
| --- | --- | --- | --- | --- | --- |
| OMP (no Apes) on OMP | OAP (100%) on OAP | 0.929 | 0.876 | 0.053 | 0 |
| OMP (no Apes) on OMP | OAP (90%) on OAP | 0.929 | 0.886 | 0.043 | 0 |
| OMP (no Apes) on OMP | OAP (70%) on OAP | 0.929 | 0.878 | 0.051 | 0 |
| OMP (no Apes) on OMP | OAP (50%) on OAP | 0.929 | 0.776 | 0.154 | 0 |
| OMP (no Apes) on OMP | OAP (30%) on OAP | 0.929 | 0.747 | 0.182 | 0 |
| OMP (no Apes) on OMP | OAP (10%) on OAP | 0.929 | 0.617 | 0.313 | 0 |
| OMP (no Apes) on OMP | OAP (100%) on OMP | 0.929 | 0.587 | 0.342 | 0 |
| OMP (no Apes) on OMP | OMP (no Apes) on OAP | 0.929 | 0.584 | 0.345 | 0 |
| OMP (no Apes) on OMP | COCO on OMP | 0.929 | 0.332 | 0.597 | 0 |
| OMP (no Apes) on OMP | COCO on OAP | 0.929 | 0.569 | 0.361 | 0 |
| OMP (no Apes) on OMP | COCO on COCO | 0.929 | 0.962 | -0.033 | 0 |
| OAP (100%) on OAP | OAP (90%) on OAP | 0.876 | 0.886 | -0.01 | 0 |
| OAP (100%) on OAP | OAP (70%) on OAP | 0.876 | 0.878 | -0.002 | 1** |
| OAP (100%) on OAP | OAP (50%) on OAP | 0.876 | 0.776 | 0.1 | 0 |
| OAP (100%) on OAP | OAP (30%) on OAP | 0.876 | 0.747 | 0.129 | 0 |
| OAP (100%) on OAP | OAP (10%) on OAP | 0.876 | 0.617 | 0.26 | 0 |
| OAP (100%) on OAP | OAP (100%) on OMP | 0.876 | 0.587 | 0.289 | 0 |
| OAP (100%) on OAP | OMP (no Apes) on OAP | 0.876 | 0.584 | 0.292 | 0 |
| OAP (100%) on OAP | COCO on OMP | 0.876 | 0.332 | 0.544 | 0 |
| OAP (100%) on OAP | COCO on OAP | 0.876 | 0.569 | 0.307 | 0 |
| OAP (100%) on OAP | COCO on COCO | 0.876 | 0.962 | -0.086 | 0 |
| OAP (90%) on OAP | OAP (70%) on OAP | 0.886 | 0.878 | 0.008 | 0 |
| OAP (90%) on OAP | OAP (50%) on OAP | 0.886 | 0.776 | 0.11 | 0 |
| OAP (90%) on OAP | OAP (30%) on OAP | 0.886 | 0.747 | 0.139 | 0 |
| OAP (90%) on OAP | OAP (10%) on OAP | 0.886 | 0.617 | 0.27 | 0 |
| OAP (90%) on OAP | OAP (100%) on OMP | 0.886 | 0.587 | 0.299 | 0 |
| OAP (90%) on OAP | OMP (no Apes) on OAP | 0.886 | 0.584 | 0.302 | 0 |
| OAP (90%) on OAP | COCO on OMP | 0.886 | 0.332 | 0.554 | 0 |
| OAP (90%) on OAP | COCO on OAP | 0.886 | 0.569 | 0.317 | 0 |
| OAP (90%) on OAP | COCO on COCO | 0.886 | 0.962 | -0.076 | 0 |
| OAP (70%) on OAP | OAP (50%) on OAP | 0.878 | 0.776 | 0.103 | 0 |
| OAP (70%) on OAP | OAP (30%) on OAP | 0.878 | 0.747 | 0.131 | 0 |
| OAP (70%) on OAP | OAP (10%) on OAP | 0.878 | 0.617 | 0.262 | 0 |
| OAP (70%) on OAP | OAP (100%) on OMP | 0.878 | 0.587 | 0.291 | 0 |
| OAP (70%) on OAP | OMP (no Apes) on OAP | 0.878 | 0.584 | 0.294 | 0 |
| OAP (70%) on OAP | COCO on OMP | 0.878 | 0.332 | 0.546 | 0 |
| OAP (70%) on OAP | COCO on OAP | 0.878 | 0.569 | 0.31 | 0 |
| OAP (70%) on OAP | COCO on COCO | 0.878 | 0.962 | -0.084 | 0 |
| OAP (50%) on OAP | OAP (30%) on OAP | 0.776 | 0.747 | 0.029 | 0 |
| OAP (50%) on OAP | OAP (10%) on OAP | 0.776 | 0.617 | 0.159 | 0 |
| OAP (50%) on OAP | OAP (100%) on OMP | 0.776 | 0.587 | 0.189 | 0 |
| OAP (50%) on OAP | OMP (no Apes) on OAP | 0.776 | 0.584 | 0.191 | 0 |
| OAP (50%) on OAP | COCO on OMP | 0.776 | 0.332 | 0.443 | 0 |
| OAP (50%) on OAP | COCO on OAP | 0.776 | 0.569 | 0.207 | 0 |
| OAP (50%) on OAP | COCO on COCO | 0.776 | 0.962 | -0.187 | 0 |
| OAP (30%) on OAP | OAP (10%) on OAP | 0.747 | 0.617 | 0.131 | 0 |
| OAP (30%) on OAP | OAP (100%) on OMP | 0.747 | 0.587 | 0.16 | 0 |
| OAP (30%) on OAP | OMP (no Apes) on OAP | 0.747 | 0.584 | 0.163 | 0 |
| OAP (30%) on OAP | COCO on OMP | 0.747 | 0.332 | 0.415 | 0 |
| OAP (30%) on OAP | COCO on OAP | 0.747 | 0.569 | 0.178 | 0 |
| OAP (30%) on OAP | COCO on COCO | 0.747 | 0.962 | -0.215 | 0 |
| OAP (10%) on OAP | OAP (100%) on OMP | 0.617 | 0.587 | 0.03 | 0 |
| OAP (10%) on OAP | OMP (no Apes) on OAP | 0.617 | 0.584 | 0.032 | 0 |
| OAP (10%) on OAP | COCO on OMP | 0.617 | 0.332 | 0.284 | 0 |
| OAP (10%) on OAP | COCO on OAP | 0.617 | 0.569 | 0.048 | 0 |
| OAP (10%) on OAP | COCO on COCO | 0.617 | 0.962 | -0.346 | 0 |
| OAP (100%) on OMP | OMP (no Apes) on OAP | 0.587 | 0.584 | 0.003 | 1** |
| OAP (100%) on OMP | COCO on OMP | 0.587 | 0.332 | 0.255 | 0 |
| OAP (100%) on OMP | COCO on OAP | 0.587 | 0.569 | 0.018 | 0 |
| OAP (100%) on OMP | COCO on COCO | 0.587 | 0.962 | -0.375 | 0 |
| OMP (no Apes) on OAP | COCO on OMP | 0.584 | 0.332 | 0.252 | 0 |
| OMP (no Apes) on OAP | COCO on OAP | 0.584 | 0.569 | 0.016 | 0 |
| OMP (no Apes) on OAP | COCO on COCO | 0.584 | 0.962 | -0.378 | 0 |
| COCO on OMP | COCO on OAP | 0.332 | 0.569 | -0.236 | 0 |
| COCO on OMP | COCO on COCO | 0.332 | 0.962 | -0.63 | 0 |
| COCO on OAP | COCO on COCO | 0.569 | 0.962 | -0.394 | 0 |
