# Supplementary material for: OpenApePose, a database of annotated ape photographs for pose estimation
Source: eLife. 2023 Dec 11;12:RP86873. doi: 10.7554/eLife.86873 (PMC10712952; doi:10.7554/eLife.86873)

OPEN MONKEY STUDIO

OpenMonkeyWild Photograph Rubric

OpenMonkeyWild *is a single-camera primate tracking system currently in development. We are currently looking to generate a very large database of images. This document lists the desiderata for that project.*

We are looking for photos to train our network. Our ultimate goal is >>50,000 images. (The precise number we need is an empirical question whose answer is not yet determined). Regardless, the key variable is variety of poses. More variety means we need fewer overall images.

Photos should meet the following requirements:

- The subject should cover at least 300x300 pixels after cropping
- The subject's entire body should be visible (no obstructions)
- Image is not blurry
- Video frames are fine but not all frames can be counted because of inherently redundancy.
- More than one monkey is fine.

Desired attributes include:

- Variety in identity
- Variety in age
- Variety in pose
- Variety in viewpoint
- Variety in background environment
- Variety in species

Some examples that are not good:

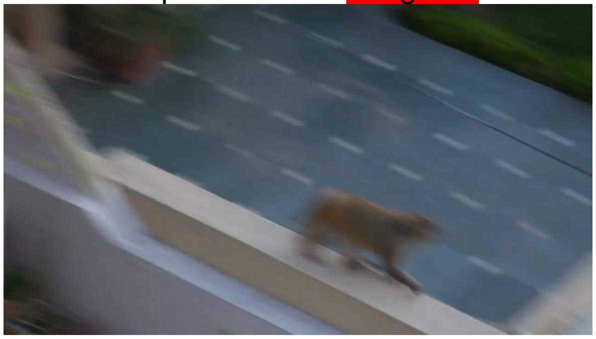

Too blurry

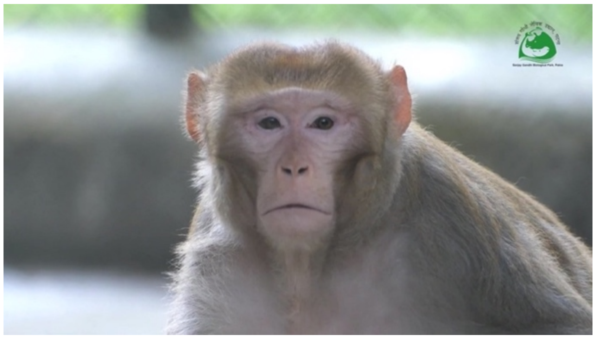

Not full body

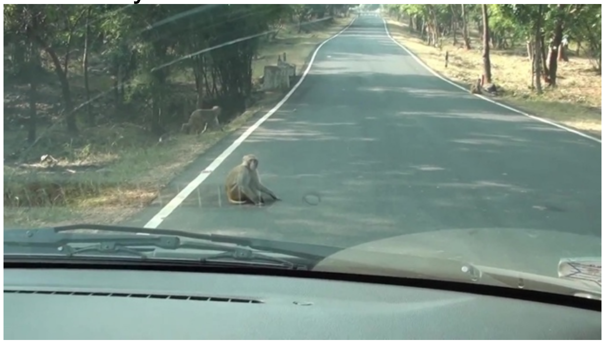

Too small

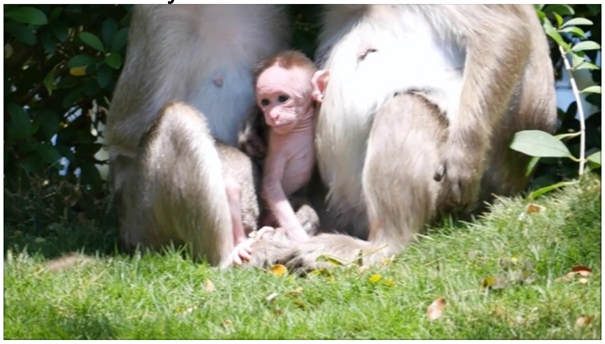

Not full body

Some examples that are good:

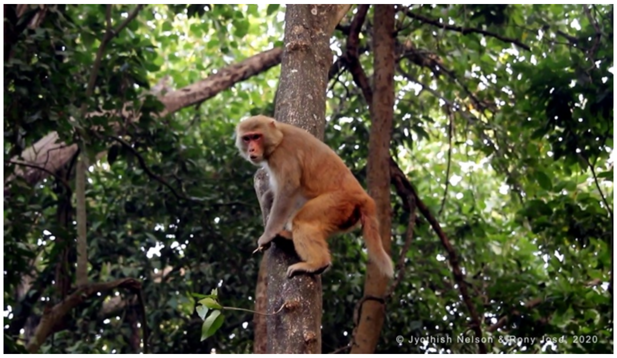

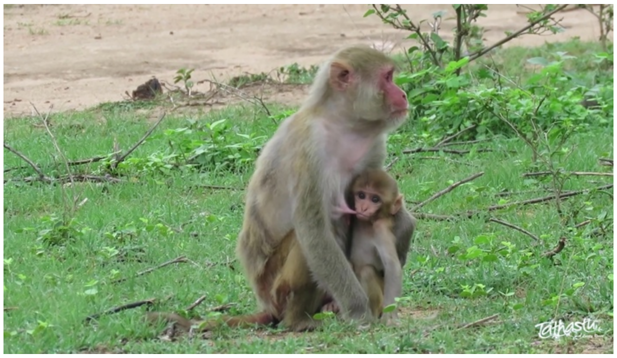

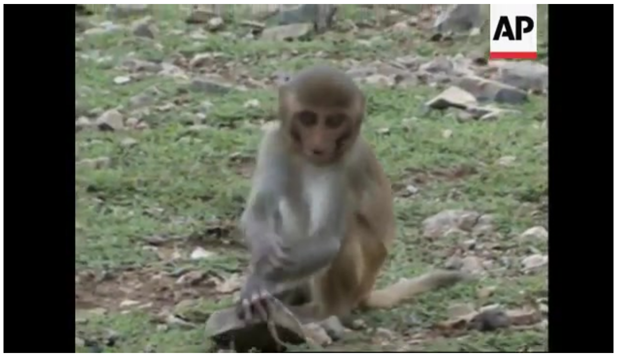

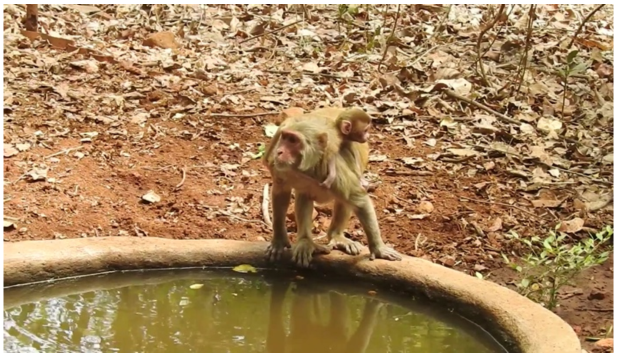

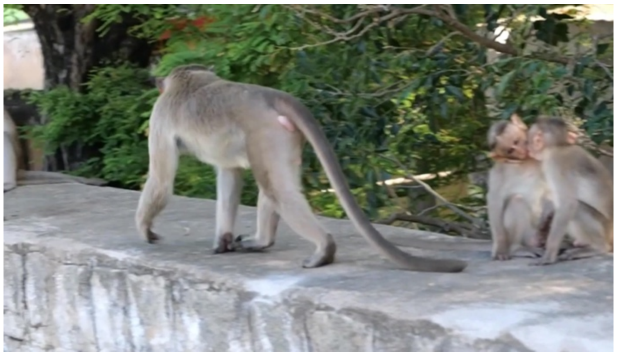

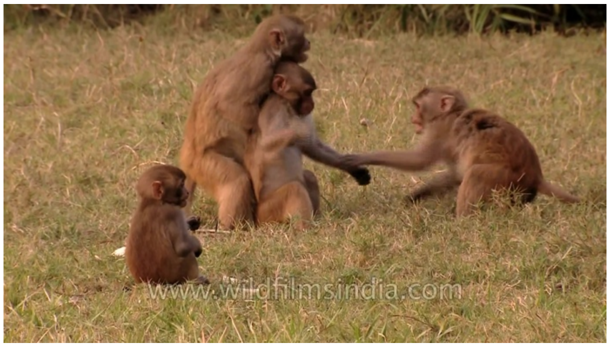

Supplement: Supplementary file 3. [file elife-86873-supp3.pdf]
